# Eupatilin-derived compounds enhance antitumor activity and doxorubicin efficacy in triple-negative breast cancer

**DOI:** 10.3389/fonc.2026.1854058

**Published:** 2026-08-12

**Authors:** Choong-Jae Lee, Jinyoung Park, Yanghee Choi, Sung Hoon Sim, Sun-Young Kong

**Affiliations:** 1 Targeted Therapy Branch, National Cancer Center, Goyang, Republic of Korea; 2 Cancer Outcome & Quality Improvement Branch, National Cancer Center, Goyang, Republic of Korea; 3 Division of Rare and Refractory Cancer, Research Institute, National Cancer Center, Goyang, Republic of Korea; 4 Department of Cancer Biomedical Science, National Cancer Center Graduate School of Cancer Science and Policy, Goyang, Republic of Korea; 5 Department of Laboratory Medicine, National Cancer Center, Goyang, Republic of Korea

**Keywords:** drug synergism, eupatilin, therapeutic efficacy, triple-negative breast cancer, xenograft model

## Abstract

**Purpose:**

Triple-negative breast cancer (TNBC) has limited therapeutic options. We evaluated the anticancer activities of the Eupatilin derivatives ONG41008 and ONG41003 and their potential to enhance doxorubicin efficacy.

**Materials and methods:**

Anticancer activity was evaluated in TNBC cells using viability, cell-cycle, apoptosis, migration, and invasion assays. Drug interactions with doxorubicin and paclitaxel were assessed using dose–response curves and the Loewe model. Doxorubicin accumulation and ABCB1 and ABCG2 mRNA expression were analyzed. ONG41003 efficacy was further evaluated in an orthotopic TNBC xenograft model.

**Results:**

Both derivatives induced G2/M arrest and apoptosis and inhibited migration and invasion. ONG41008 showed an apparent reduction in the fitted doxorubicin IC_50_ at 60 μM and clear synergy with doxorubicin. ONG41003 did not consistently reduce the doxorubicin IC_50_ and showed a modest positive interaction. Neither compound showed meaningful synergy with paclitaxel. Combination treatment increased doxorubicin accumulation and attenuated doxorubicin-induced ABCB1 expression. *In vivo*, ONG41003 enhanced doxorubicin efficacy without detectable systemic toxicity.

**Conclusion:**

ONG41008 and ONG41003 exerted anticancer effects and enhanced the response to doxorubicin through distinct interactions. Increased doxorubicin accumulation may be associated with attenuation of ABCB1 expression, supporting further investigation of these compounds as partners for anthracycline-based chemotherapy.

## 1 Introduction

Breast cancer is the most commonly diagnosed cancer and leading cause of cancer death in women (1). Triple-negative breast cancer (TNBC), which accounts for 15–20% of all breast cancer cases, lacks the expression of the estrogen receptor (ER), progesterone receptor (PR), and human epidermal growth factor receptor-2 (HER2) (2). TNBC is the most aggressive subtype of breast cancer with high invasion capability, metastatic potential, recurrence rate and poor clinical outcome (3). 10-year recurrence free survival (RFS) rate for early-stage TNBC is below 70%. Furthermore, advanced TNBC showed that the duration of response for first-line chemotherapy is less than 6 months and the average overall survival is only 1–2 years (4). Despite recent advances, current therapeutic strategies for TNBC largely rely on cytotoxic chemotherapy and are limited by rapid acquisition of drug resistance and pronounced tumor heterogeneity. Although immune checkpoint inhibitors and targeted therapies have shown clinical benefit in selected patient subsets, the proportion of responders remains modest and robust predictive biomarkers are still lacking. Consequently, there is a critical unmet need to elucidate the molecular mechanisms underlying TNBC aggressiveness and therapeutic resistance and to develop novel therapeutic strategies that can improve durable clinical outcomes for patients with TNBC.

Eupatilin (5,7-dihydroxy-3′,4′,6-trimethoxyflavone) is a naturally occurring flavonoid predominantly isolated from artemisia (*Artemisia asiatica* Nakai and *Artemisia princeps* Pampanini), a medicinal herb traditionally used in Asian medicine. It has been widely recognized for its gastroprotective properties and is clinically used for the treatment of gastric mucosal injury and inflammatory gastrointestinal disorders. Beyond its original therapeutic indications, Eupatilin has been reported to have various biologic activities, including anti-ulcerative, anti-inflammatory, and anti-oxidative effects, which are largely attributed to its ability to modulate oxidative stress and inflammatory signaling pathways (5, 6). Owing to these properties, Eupatilin has been investigated in various disease contexts, such as inflammatory bowel disease, hepatic injury, and neuroinflammatory conditions.

More recently, accumulating evidence has suggested that Eupatilin also exerts anti-cancer effects across multiple tumor types including gastric, colorectal, and hepatic cancers by suppressing cancer cell proliferation, migration, and survival, as well as by modulating key oncogenic signaling pathways such as PI3K/AKT and AMPK signaling pathways (7, 8). Its therapeutic potential breast cancer remains poorly defined. Moreover, despite these emerging findings, its direct clinical application as an anti-cancer agent remains limited due to modest single-agent efficacy and unfavorable pharmacokinetic properties. Its anti-tumor effects are typically observed at relatively high concentrations and are modest compared with those of conventional cytotoxic agents. These limitations suggest that Eupatilin alone is unlikely to induce robust and durable tumor suppression in aggressive cancers such as TNBC, thereby underscoring the need for molecular optimization or structural modification of Eupatilin to enhance its anti-cancer efficacy. Previous studies have suggested that ONG41008, a synthetic polyphenol derivative of Eupatilin, exhibits anti-fibrotic and anti-cancer activities in pancreatic ductal adenocarcinoma and lung adenocarcinoma models through the induction of senescence-coupled apoptosis and the modulation of tumor-associated signaling pathways. However, its efficacy in TNBC models remains unexplored (9).

In this study, we introduce two Eupatilin derivatives, ONG41008 and ONG41003, and systematically evaluate their anti-tumor activities in TNBC using *in vitro* and *in vivo* models. In addition, we investigated their potential to enhance the therapeutic efficacy of doxorubicin and explored the mechanisms underlying their combinatorial effects. Through these studies, we sought to assess the therapeutic potential of Eupatilin derivatives as novel therapeutic candidates and combination partners for TNBC.

## 2 Materials and methods

### 2.1 Cell lines and chemicals

Cell lines (MDA-MB-231, MDA-MB-453) were purchased from the Korea Cell Line Bank (KCLB, Seoul, Korea) and HCC-70 was obtained from the American Type Culture Collection (ATCC, Manassas, VA, USA). Cell lines were maintained in appropriate culture media: MDA-MB-453 in RPMI-1640 medium (Gibco, Grand Island, NY, USA) and MDA-MB-231 in Dulbecco’s Modified Eagle Medium (Gibco). All culture media were supplemented with 10% fetal bovine serum and 1% penicillin/streptomycin (Gibco). Cells were cultured in a humidified incubator at 37 °C with 5% CO_2_. For experimental procedures, cells were detached using 1× trypsin/EDTA solution, and cell viability and counting were performed using the EVE™ Automated Cell Counter (NanoEntek, Seoul, Korea). ONG41008 and ONG41003 were obtained from Osteoneurogen Inc. (Seoul, Republic of Korea), and dissolved in dimethyl sulfoxide (DMSO, Sigma, St. Louis, MO, USA) for *in vitro* studies or in 4% w/v of gelucire 44/14 (Sigma) for animal study.

### 2.2 Drug cytotoxicity and synergistic effect

For assessing cytotoxicity of ONG41008 and ONG41003 at single dose, 2 x 10^4^ cells/well were seeded in 96-well plates and incubated for 24 h to allow cell attachment. Subsequently, cells were treated for 72 h with ONG41008 and ONG41003 at serially diluted concentrations. Cell viability was assessed using the CellTiter-Glo^®^ 2.0 assay (Promega, Madison, WI, USA), and luminescence signals were measured as relative luminescence units (RLU) using the Infinite 200 Pro microplate reader (Tecan, Männedorf, Switzerland). The half-maximal inhibitory concentration (IC_50_) for each drug was calculated by nonlinear regression of the dose–response curves using GraphPaf Prism (version 8.3.1).

For evaluation of Synergistic effect, we assessed the cytotoxicity when these drugs were combined with the standard anti-cancer drugs doxorubicin (Chemscene, Monmouth Junction, NJ, USA) and paclitaxel (Chemscene). Cell lines were seeded in a 384 well plate with 800 cells in 20 uL complete media in each well. After 72h, cell viability was evaluated by luminescence using CellTiter-Glo^®^ 2.0 cell viability assay reagent (Promega). To quantify the synergistic effect of the test compounds, Loewe scoring model was also used to calculate the expected responses of the combination treatment of ONG41008 and ONG41003 with doxorubicin using SynergyFinder 2.0 software (https://synergyfinder.fimm.fi) (10). SynergyFinder 2.0 is a web application for interactive analysis and visualization of two or more drug combination screening data.

### 2.3 Cell proliferation assay

2 x 10^4^ cells/well were seeded in 96-well plates with drug treatment and incubated in IncuCyte (Sartorius) for three days. Cell confluency was assessed using Incucyte S3 Software v2018B (Satorius).

### 2.4 Cell cycle assay

Cell cycle analysis was performed using propidium iodide (PI; Sigma). After Eupatilin, ONG41008 and ONG41003 treatment, the cells were collected and fixed in cold 70% ethanol for 4 h at −20 °C. Cells were then washed once with phosphate-buffered saline (PBS) and incubated with PI staining solution (50 μg/ml PI, 100 μg/ml RNase) for 30 min at room temperature. Cell cycle analysis was carried out using flow cytometry (BD FACSLyric™, BD Biosciences, San Jose, CA, USA). At least 10,000 single cells were analyzed.

### 2.5 Annexin V apoptosis assay

Quantitative analysis of the apoptotic cells was performed using the eBioscience™ Annexin V Apoptosis Detection Kits (Invitrogen, Carlsbad, MA, USA). After Eupatilin, ONG41008 and ONG41003 treatment, the cells were collected and washed twice with cold PBS. The cells were resuspended in 100 μl of binding buffer (1x10^5^ cells) and stained with 5 μl of FITC Annexin V and propidium iodide (PI). Then, the mixture was incubated at room temperature for 15 min in the dark. After incubation, 400 μl of binding buffer was added, and the samples were then analyzed by BD FACSLyric™ (BD Biosciences).

### 2.6 Invasion assay

THINCERT cell culture insert for 24 well plates (Greiner Bio-one, Frickenhausen, Germany) were employed for invasion assay. 1 x 10^5^ cells/well were seeded on the upper chamber of culture inserts after coating with 250 μg/mL Matrigel (Corning, Glendale, AZ, USA) in serum-free medium with or without Eupatilin, ONG41008 and ONG41003. The bottom chambers were filled with medium supplemented with 10% fetal bovine serum. After incubation for 24 h at 37 °C, the cells that invaded through to the bottom of the insert membrane were fixed, stained with wright-giemsa, and counted the cells selected 6 random regions under a phase-contrast microscope (Carl Zeiss, Oberkochen, Germany).

### 2.7 Wound-healing assay

Cells were seeded in 96-well plate. After incubation for 24 h at 37 °C, we scrape the cell monolayer in a straight line with a 200 μL pipette tip and remove debris by washing with PBS. Cells were treated Eupatilin, ONG41008 and ONG41003 after wound scratch and incubated for 24 h. Phase-contrast images of cells were captured using a Sartorius IncuCyte^®^ S3 system (Satorius) at a total magnification of 100x. The wound area at time zero or the endpoint was measured using Incucyte S3 Software v2018B. The area of wound closure was calculated as a percentage of the initial wound area.

### 2.8 Western blot

Cells were lysed with radio-immunoprecipitation buffer (50 mM Tris-Cl, pH 7.5, 150 mM NaCl, 0.1% SDS, 0.5% sodium deoxycholate and 1 mM EDTA) containing protease and phosphatase inhibitors (1 mM NaF, 1 mM Na_3_OV_4_, PMSF, 2 mg/ml leupeptin and pepstatin; all purchased from Sigma) for 30 min at 4 °C. The protein concentration was quantified with a Pierce BCA protein assay kit (Thermo Scientific). Blots were incubated with primary antibodies at 4 °C overnight. The following antibodies were used for immunoblots: anti-Caspase-3 (Cell Signaling #9662, 1:1,000), anti-PARP (Cell Signaling #5625, 1:1,000), anti-AKT (Cell Signaling #9272, 1:1,000), anti-phopho-AKT (Cell Signaling #9271, 1:1,000), anti-PI3K (Cell Signaling #4257, 1:1,000), anti-phospho-PI3K (Cell Signaling #17366, 1:1,000), anti-AMPK (Cell Signaling #2795, 1:1,000), anti-phospho-AMPK (Cell Signaling #2537, 1:1,000) and anti-β Actin (INVITROGEN #MA1-140, 1:5,000).

### 2.9 Gene expression analysis by RT-qPCR

Cells were seeded in 12-well plates and incubated for 24 h. Cells were then treated with ONG41008 or ONG41003 for 24 h. Total RNA was extracted using the AllPrep kit (QIAGEN, Hilden, Germany) and complementary DNA was synthesized from 1 μg of total RNA with the First Strand cDNA Synthesis Kit (Roche, Basel, Switzerland) following the manufacturer’s protocols. Real-time quantitative PCR was performed using the LightCycler^®^ Real-Time PCR system (Roche) with FastStart Essential DNA Green Master (Roche). Gene expression levels were calculated using the DDCt (Delta–Delta Ct) method, with GAPDH serving as the endogenous control. The sequences of the primers used in this study are listed below.

### 2.10 Animal study

All mice used in this study were housed under specific pathogen-free conditions and cared for in accordance with the Institutional Animal Care and Use Committee (IACUC) at National Cancer Center, South Korea (No. NCC-24-981). To evaluate the anti-cancer effects of ONG41003 and its Synergistic effect with doxorubicin, we used a xenograft model. MDA-MB-231 cells (1 x 10^7^ cells/mouse mixed with Matrigel) were injected into Balb/c nude mouse. After tumor cell injection, when the tumor volume reached approximately 150 mm^3^, the mice were randomly grouped and injected with drugs. ONG41003 was dissolved in a vehicle consisting of 2.5% (w/v) citric acid and 4% (w/v) Gelucire 44/14 in distilled water for *in vivo* study. The tumor size was measured daily, and the tumor volume was calculated with the following formula: volume = (longitudinal x transverse^2^)/2.

### 2.11 Immunohistochemistry

Samples were fixed with 4% paraformaldehyde and embedded with paraffin for Immunohistochemistry staining. Paraffin-embedded tissue blocks were manually sectioned with a microtome to obtain 4 μm thick sections. Paraffin sections were dewaxed and stained with hematoxylin (Dako, Carpinteria, CA, USA) and eosin (Millipore, Billerica, MD, USA) according to the supplier’s instructions. Target proteins were visualized by immunohistochemistry (IHC). Staining was performed using primary anti-Ki67 (Abcam, Cambridge, MA, USA) and anti-Caspase 3 (Cell Signaling Technology, Danvers, MA, USA) antibodies. Nuclei were counterstained with hematoxylin (Dako, Carpinteria, CA, USA) for IHC. Secondary antibodies conjugated with horseradish peroxidase (HRP, Dako) were used to visualize target proteins. Target proteins were visualized by the DAB reaction and imaged using Vectra Polaris system (Akoya Biosciences, Marlborough, MA, USA). DAB intensity was automatically quantified with InForm software (Akoya) in 3 random spots per every tissue sample.

### 2.12 Accumulation of doxorubicin *in vitro* and *in vivo*

To evaluate intracellular and intratumoral accumulation of doxorubicin, MDA-MB-231 cells (1 × 10^5 cells/well) were seeded in 24-well plates and incubated for 24 h. Cells were treated with doxorubicin alone or in combination with ONG41008 or ONG41003. After 1, 2, 4, and 6 h of treatment, cells were fixed with 4% paraformaldehyde for 20 min at room temperature, washed with PBS, and counterstained with DAPI (Invitrogen, Carlsbad, CA, USA). For the *in vivo* study, Doxorubicin-associated fluorescence was examined in unstained adjacent tumor sections. The fluorescence of doxorubicin was visualized using a ZEISS fluorescence microscope with ZEN 3.10 software under identical acquisition settings. Images were acquired using identical microscope settings and exposure time. A common min/max display range was established in ZEN software using the doxorubicin-only group and applied unchanged to the combination group.

### 2.13 Statistics

All statistical data are expressed as the mean ± standard deviation (SD) (n=3). Statistical comparisons were determined by Student’s t-test between two groups or by one-way ANOVA with Dunnett’s multiple comparison among more than three groups. In the case of *in vivo* analysis, the number of mice used in the experiments was indicated in the legends. Asterisks are used to indicate statistical significance. *, **, and *** indicate p < 0.05, p < 0.01, and p < 0.001, respectively.

## 3 Results

### 3.1 Structural modification of Eupatilin generated the derivatives ONG41008 and ONG41003

**Figure 1:**
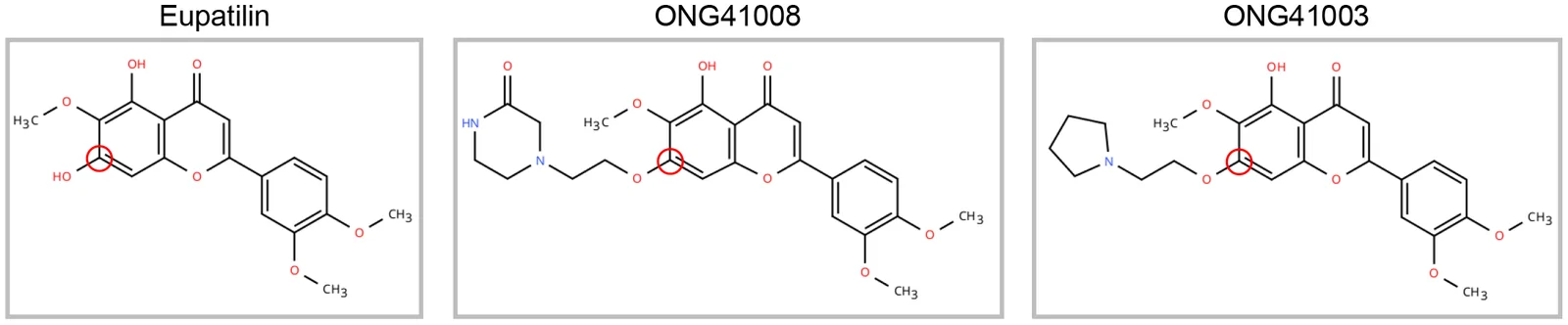
Structure of Eupatilin, ONG41008, and ONG41003. Chemical structures of Eupatilin, ONG41008, and ONG41003 are shown. ONG41008 and ONG41003 were synthesized by introducing distinct amine-containing alkoxy side chains at specific glucuronidation sites of the Eupatilin scaffold to improve metabolic stability and physicochemical properties.

ONG41008 and ONG41003 were generated as semi-synthetic Eupatilin derivatives, by introducing amine-containing alkoxy side chains at a specific phenolic position of the flavone scaffold (**Figure 1**). These derivatives retain the core flavone structure of Eupatilin while incorporating additional chemical moieties commonly employed to modulate physicochemical and pharmacological properties.

### 3.2 ONG41008 and ONG41003 exhibits preferential cytotoxicity toward TNBC cells

**Figure 2:**
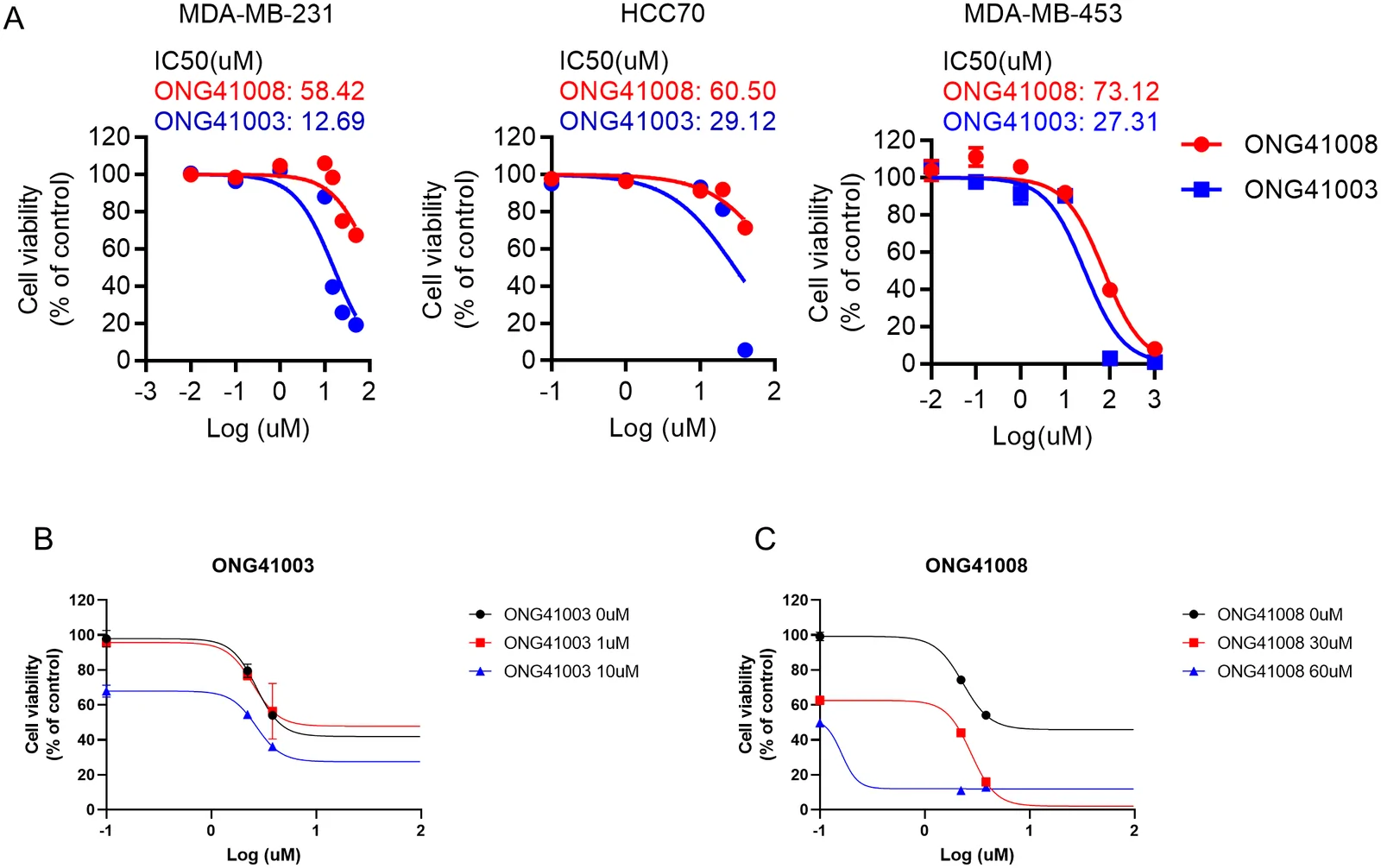
ONG41008 and ONG41003 decreased cell viability and sensitized TNBC cells to doxorubicin. **(A)** Cell viability of MDA-MB-231, HCC70, and MDA-MB-453 cells was evaluated following treatment with serially diluted concentrations of ONG41008 or ONG41003 for 72 h using the CellTiter-Glo^®^ assay. Dose-response curves were generated, and IC50 values were calculated for each compound. **(B, C)** Doxorubicin dose–response curves generated in MDA-MB-231 cells in the presence of fixed concentrations of ONG41003 (B; 0, 1, or 10 μM) or ONG41008 (C; 0, 30, or 60 μM). The fitted doxorubicin IC_50_ values were 2.698, 2.502, and 2.683 μM with increasing ONG41003 concentrations and 2.310, 2.765, and 0.1617 μM with increasing ONG41008 concentrations. All nonlinear fits were classified as ambiguous. Data are presented as the mean ± SD of three independent experiments.

To evaluate the cytotoxic effect of ONG41008 and ONG41003 on TNBC cells, cell viability was assessed across a range of serially diluted concentrations in three TNBC cells (MDA-MB-231, MDA-MB-453, and HCC-70). ONG41008 exhibited minimal cytotoxicity, with high IC_50_ values across all tested cell lines (**Figure 2A**). In contrast, ONG41003 consistently reduced cell viability in all TNBC cell lines examined, indicating a broad antitumor activity (**Figure 2A**). We next generated doxorubicin dose–response curves in the presence of fixed concentrations of each compound. The fitted doxorubicin IC_50_ values were approximately 2.698, 2.502, and 2.683 μM in the 0, 1, and 10 μM ONG41003, respectively, indicating no consistent IC_50_ reduction. In the 0, 30, and 60 μM ONG41008, the fitted IC_50_ values were approximately 2.310, 2.765, and 0.1617 μM, respectively. Thus, an apparent IC_50_ reduction was observed only at 60 μM ONG41008 (**Figures 2B, C**). Because the nonlinear fits were ambiguous and the compounds affected cell viability as single agents, drug synergy was evaluated separately using dose–response matrices and the Loewe model.

### 3.3 ONG41008 and ONG41003 suppress TNBC cell growth through induction of G2/M cell cycle arrest and apoptosis

**Figure 3:**
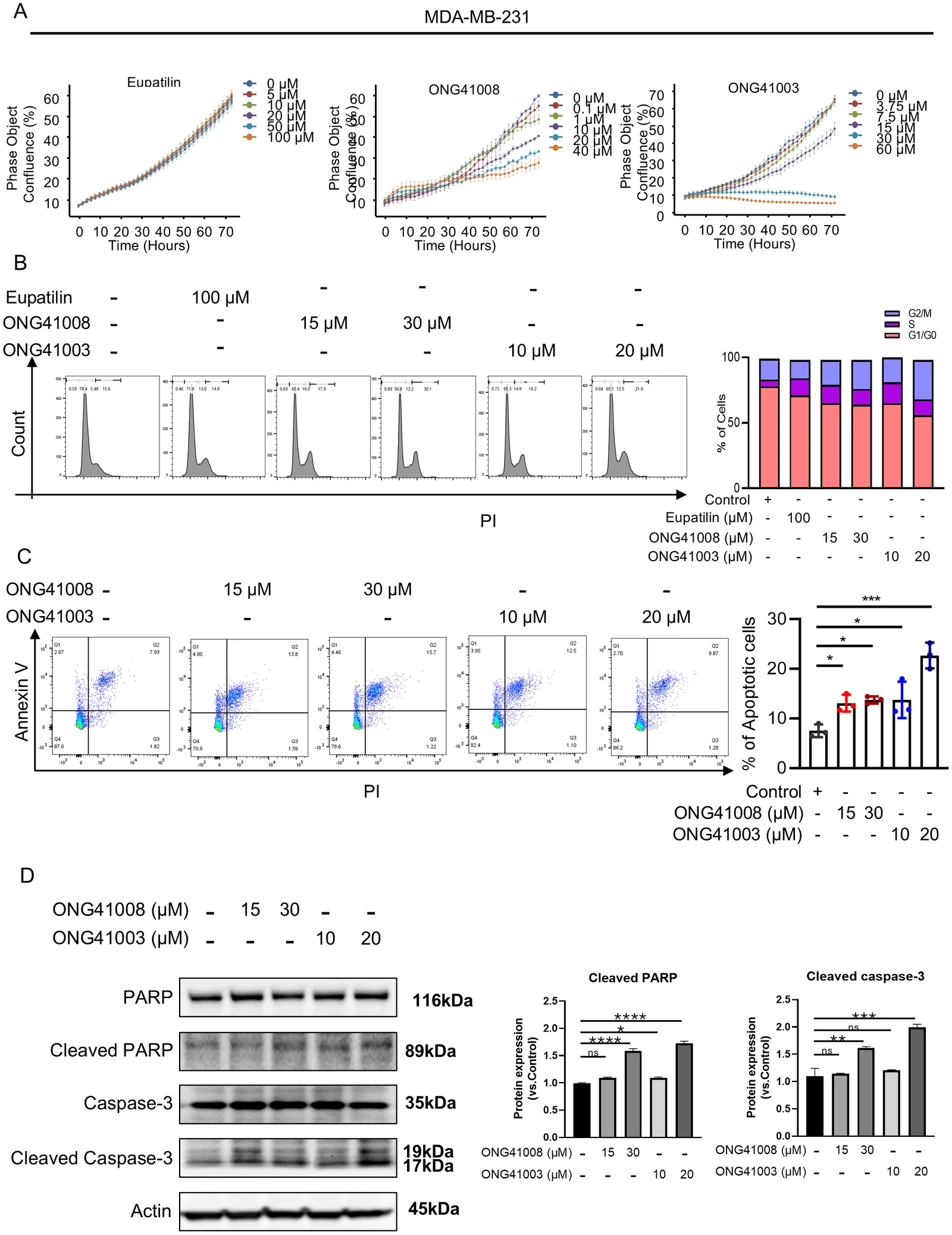
The effects of ONG41008 and ONG41003 on the cell growth and apoptosis in MDA-MB-231 cells. **(A)** Real-time proliferation analysis of MDA-MB-231 cells treated with the indicated concentrations of Eupatilin, ONG41008 or ONG41003. Cell confluency was monitored using the IncuCyte^®^ live-cell imaging system for 72 h. **(B)** Cell cycle analysis following treatment with of Eupatilin, ONG41008 or ONG41003. Cells were stained with propidium iodide (PI) and analyzed by flow cytometry. Representative histograms and quantification of cell populations in G1/G0, S, and G2/M phases are shown. **(C)** Apoptosis analysis following treatment with ONG41008 or ONG41003. Cells were stained with Annexin V-FITC and PI and analyzed by flow cytometry. Representative flow cytometry plots and quantification of apoptotic cells are shown. **(D)** Western blot analysis of apoptosis-associated proteins following treatment with ONG41008 or ONG41003. Representative blot images and relative quantification of protein expression levels are shown. Actin was used as an internal loading control. Data are presented as mean ± SD from three independent experiments (n = 3). *p < 0.05, **p < 0.01, ***p < 0.001, ****p < 0.0001 versus control group.

To compare the anti-proliferative activities of the Eupatilin derivatives with the parental compound, we evaluated cell proliferation in the human TNBC cell line MDA-MB-231. While Eupatilin exhibited only modest inhibition of cell proliferation over the tested concentration range, both ONG41008 and ONG41003 markedly suppressed cell proliferation in a concentration-dependent manner, with ONG41008 showing greater potency than ONG41003 (**Figure 3A**). These findings indicate that structural modification of Eupatilin substantially enhanced its anti-proliferative activity. To investigate the mechanism underlying growth inhibition, we next examined cell cycle progression and apoptosis. Flow cytometric analysis demonstrated that treatment with ONG41008 or ONG41003 altered cell cycle distribution, characterized by a reduction in the G0/G1 population and accumulation of cells in the G2/M phase, indicating G2/M cell cycle arrest (**Figure 3B**). Consistently, Annexin V/PI staining revealed a concentration-dependent increase in apoptotic cell populations following treatment with both derivatives (**Figure 3C**). Western blot analysis further confirmed apoptosis induction. Treatment with ONG41008 (30 μM) and ONG41003 (20 μM) increased the levels of cleaved PARP and cleaved caspase-3 compared with untreated cells (**Figure 3D**). Collectively, these findings demonstrate that ONG41008 and ONG41003 inhibit TNBC cell growth by inducing G2/M cell cycle arrest and apoptosis, with ONG41008 exhibiting the greatest anti-tumor activity among the tested compounds.

### 3.4 ONG41008 and ONG41003 attenuate TNBC cell migration and invasion and are associated with changes in PI3K/AKT and AMPK signaling

**Figure 4:**
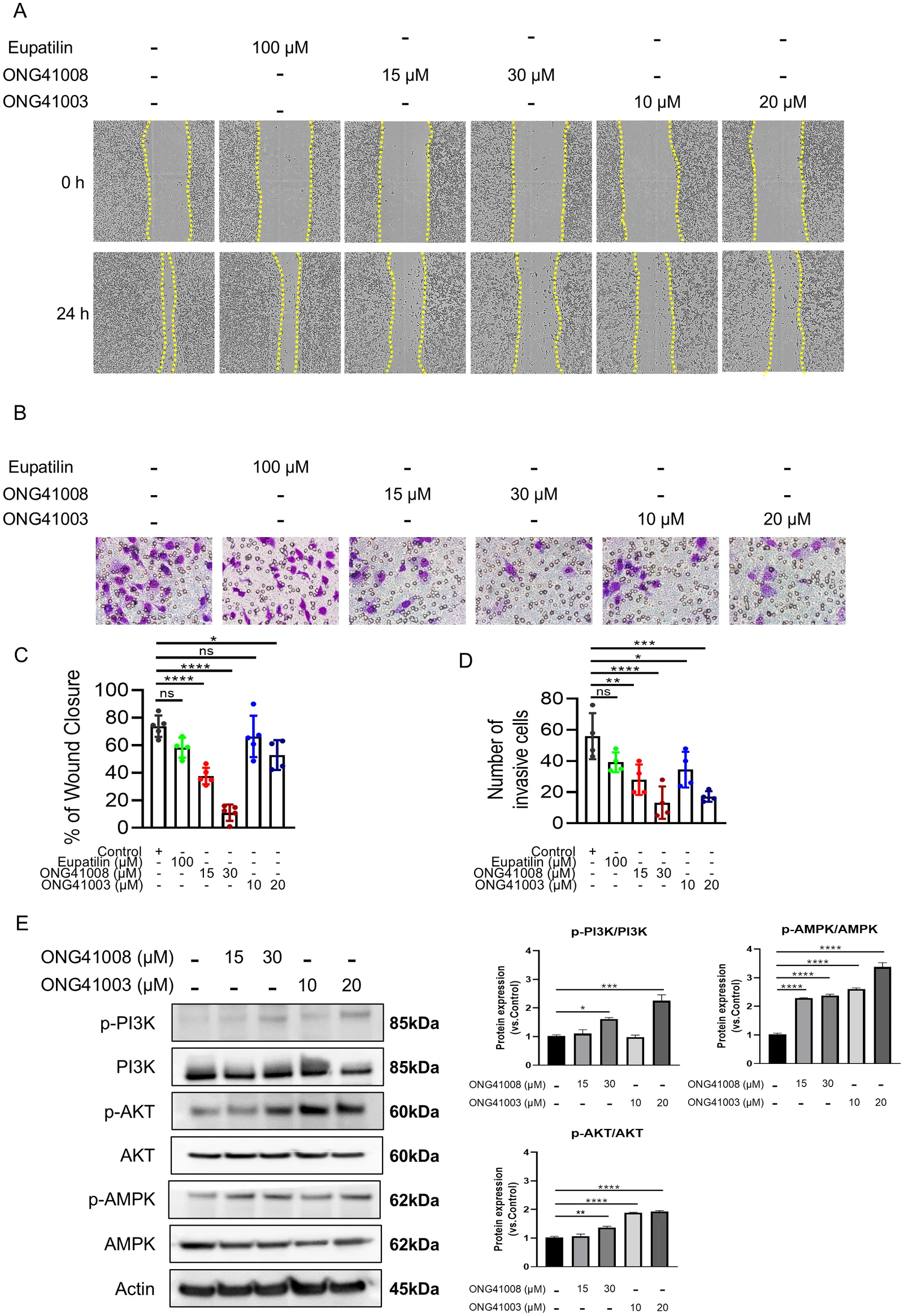
The effects of ONG41008 and ONG41003 on migratory capacity in MDA-MB-231 cells. **(A, C)** Wound-healing assay evaluating the migratory capacity of MDA-MB-231 cells following treatment with ONG41008 or ONG41003. Representative phase-contrast images were acquired at 0 h and 24 h after wound generation using the IncuCyte^®^ S3 imaging system. Quantification of wound closure was calculated as the percentage of the initial wound area. **(B, D)** Matrigel invasion assay evaluating the invasive capacity of MDA-MB-231 cells treated with ONG41008 or ONG41003. Cells invading through Matrigel-coated membranes were fixed, stained with Wright–Giemsa solution, and quantified in six randomly selected microscopic fields per insert. Representative microscopic images and quantified invasive cell counts are shown. **(E)** Western blot analysis of total and phosphorylated PI3K, AKT, and AMPK following treatment with ONG41008 or ONG41003. Representative blot images and relative quantification of protein expression levels are shown. Actin was used as an internal loading control. Data are presented as mean ± SD from three independent experiments (n = 3). ns, not significant; *p < 0.05, **p < 0.01, ***p < 0.001, ****p < 0.0001 versus control group.

TNBCs are characterized by an intrinsically aggressive phenotype, including markedly enhanced migratory and invasive capacities compared with other breast cancer subtypes (11, 12). Given the critical contribution of cell motility to metastatic dissemination, we evaluated the effects of the Eupatilin, ONG41008 and ONG41003 on TNBC cell migration and invasion. In wound-healing assays, Eupatilin produced only a modest inhibitory effect on cell migration, whereas both ONG41008 and ONG41003 inhibited migration in a concentration-dependent manner, with ONG41008 exhibiting the strongest activity (**Figures 4A, C**). Likewise, all three compounds reduced the invasive capacity of MDA-MB-231 cells, however, the anti-invasive effects of ONG41008 and ONG41003 were markedly greater than those of Eupatilin (**Figures 4B, D**). To investigate signaling pathways associated with these biological effects, the phosphorylation status of PI3K, AKT, and AMPK was analyzed by western blotting. Compared with untreated cells, treatment with ONG41008 and ONG41003 increased the p-PI3K/PI3K, p-AKT/AKT, and p-AMPK/AMPK ratios, although the magnitude of these changes differed between the two derivatives (**Figure 4E**). These findings demonstrate that ONG41008 and ONG41003 exhibit migration- and invasion-inhibitory effects activity compared with Eupatilin and are associated with altered PI3K/AKT and AMPK signaling in TNBC cells.

### 3.5 Treatment of ONG41008 or ONG41003 with doxorubicin exhibited synergistic effects

**Figure 5:**
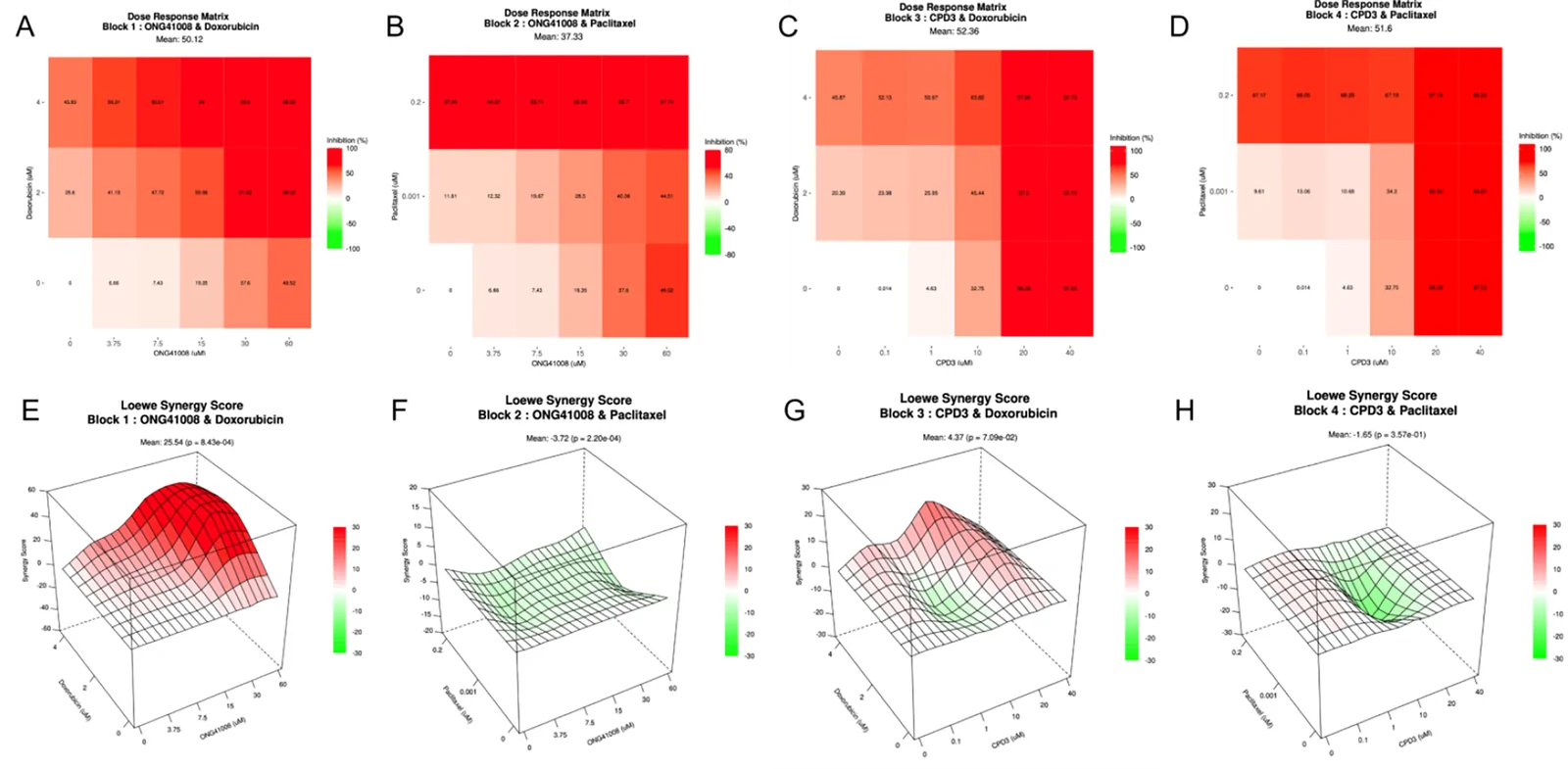
Synergistic effects of Eupatilin derivatives with doxorubicin or paclitaxel. **(A–D)** Dose-response matrices showing the percentage inhibition of cell viability in MDA-MB-231 cells treated with combinations of ONG41008 or ONG41003 and either doxorubicin or paclitaxel for 72 h. Cell viability was measured using the CellTiter-Glo^®^ assay. Values within each block represent the mean percentage inhibition relative to untreated controls. **(E–H)** Three-dimensional (3D) Loewe synergy score plots generated using SynergyFinder 2.0 software. Positive synergy scores indicate synergistic interactions, whereas negative scores indicate antagonistic or non-synergistic interactions. The z-axis represents the synergy score, whereas the x- and y-axes represent the concentration ranges of each drug combination: ONG41008 + doxorubicin **(E)**, ONG41008 + paclitaxel **(F)**, ONG41003 + doxorubicin **(G)**, and ONG41003 + paclitaxel **(H)**.

Combination therapy is widely used for the cancer treatment to enhance therapeutic efficacy through synergistic effect and to improve treatment responses (13). To quantitatively determine whether the combined treatment of ONG41008 and ONG41003 with doxorubicin or paclitaxel exerted synergism, additivity, or antagonism, Loewe scoring model were used to analyze our data via SynergyFinder 2.0 software (10, 14). Notably, the combination of ONG41008 with doxorubicin exhibited a high mean synergy score of 25.54, indicating a synergistic interaction, whereas its combination with paclitaxel resulted in a negative synergy score (−3.72), reflecting an overall antagonistic or non-synergistic effect (**Figures 5A, B, E, F**). In case of ONG41003, the combination with doxorubicin obtained a mean synergy score of 4.37, while the combination with paclitaxel exhibited score of -1.65, indicating that ONG41003 had a Synergistic effect with doxorubicin, not paclitaxel (**Figures 5C, D, G, H**). Overall, these results demonstrate that both ONG41008 and ONG41003 selectively synergize with doxorubicin, but not with paclitaxel, in TNBC cells. This drug-specific synergy highlights the importance of rational combination strategies and supports the potential of ONG41008 and ONG41003 as effective combination partners with anthracycline-based chemotherapy.

### 3.6 ONG41008 and ONG41003 enhance intracellular doxorubicin accumulation without altering ABCB1 or ABCG2 expression

**Figure 6:**
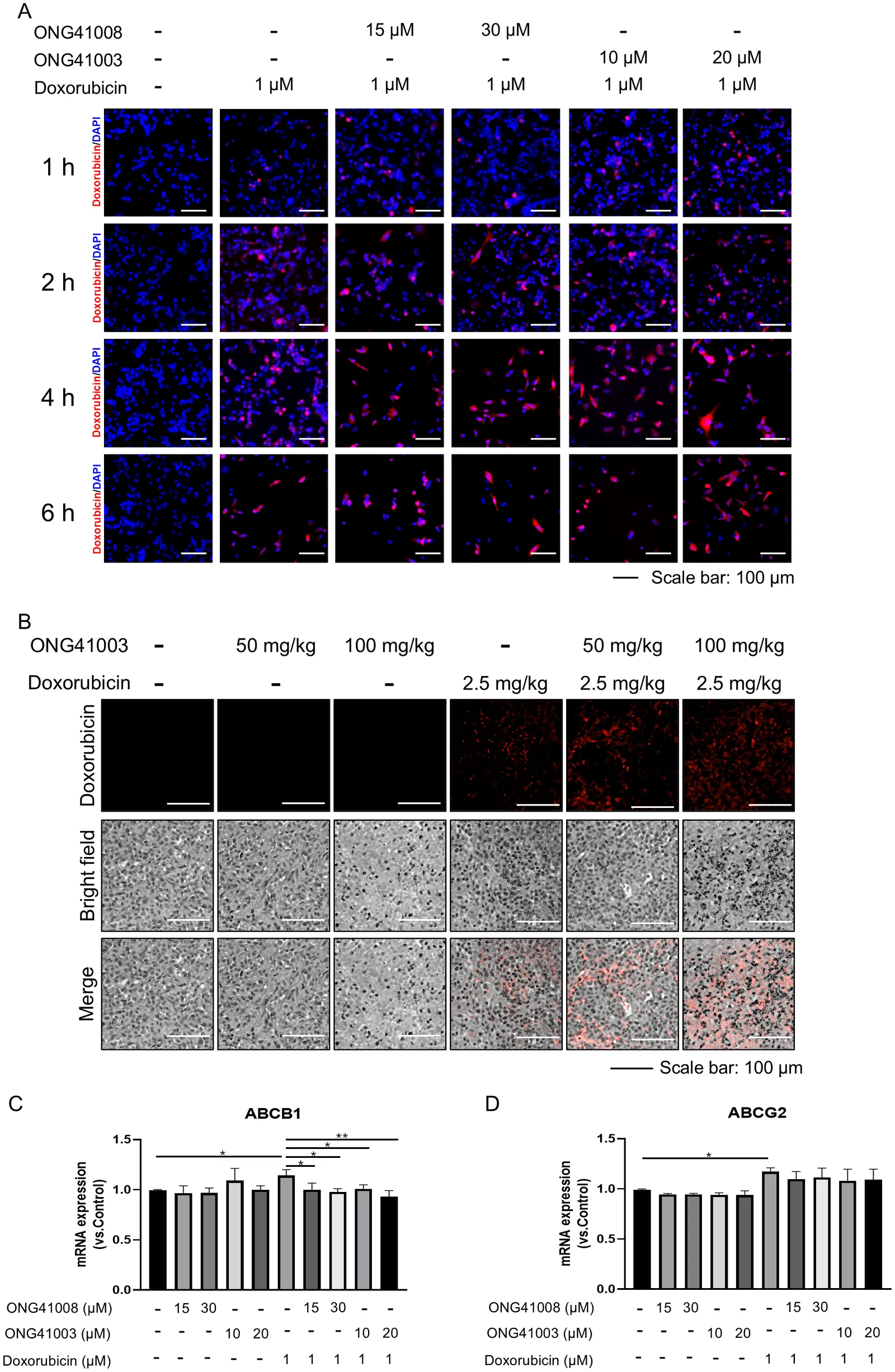
ONG41008 and ONG41003 enhance doxorubicin accumulation and attenuate doxorubicin-induced ABCB1 expression. **(A)** Representative fluorescence images of MDA-MB-231 cells treated with doxorubicin alone or in combination with ONG41008 or ONG41003. Doxorubicin fluorescence (red) and DAPI nuclear staining (blue) are shown. **(B)** Representative fluorescence images of unstained tumor sections from MDA-MB-231 xenografts treated with vehicle, doxorubicin, ONG41003, or the combination. Doxorubicin fluorescence (red) is shown. **(C, D)** mRNA expression levels of ABCB1 **(C)** and ABCG2 **(D)** following treatment with doxorubicin alone or in combination with ONG41008 or ONG41003. Data are presented as mean ± SD from three independent experiments (n = 3). *p < 0.05, **p < 0.01 versus the doxorubicin-only treated group.

To investigate the mechanism underlying the synergistic interaction between the Eupatilin derivatives and doxorubicin, we first examined intracellular doxorubicin accumulation. Fluorescence imaging demonstrated that co-treatment with either ONG41008 or ONG41003 increased intracellular doxorubicin accumulation compared with doxorubicin treatment alone in MDA-MB-231 cells, with the effect becoming more evident over time (**Figure 6A**). Consistent with the *in vitro* findings, enhanced doxorubicin fluorescence was also observed in xenograft tumor sections from mice treated with the combination therapy compared with doxorubicin alone, indicating increased intratumoral accumulation of doxorubicin *in vivo* (**Figure 6B**). To explore a potential mechanism underlying this enhanced intracellular accumulation, we examined the mRNA expression of the drug efflux transporters ABCB1 and ABCG2. Doxorubicin treatment increased the expression of both transporters, whereas co-treatment with ONG41008 or ONG41003 significantly attenuated the induction of ABCB1. In contrast, ABCG2 expression was not significantly altered by combination treatment (**Figures 6C, D**). These findings suggest that ONG41008 and ONG41003 may enhance intracellular doxorubicin accumulation, at least in part, through suppression of ABCB1-associated drug efflux.

### 3.7 Combination of ONG41003 with doxorubicin has synergistic effect *in vivo*

**Figure 7:**
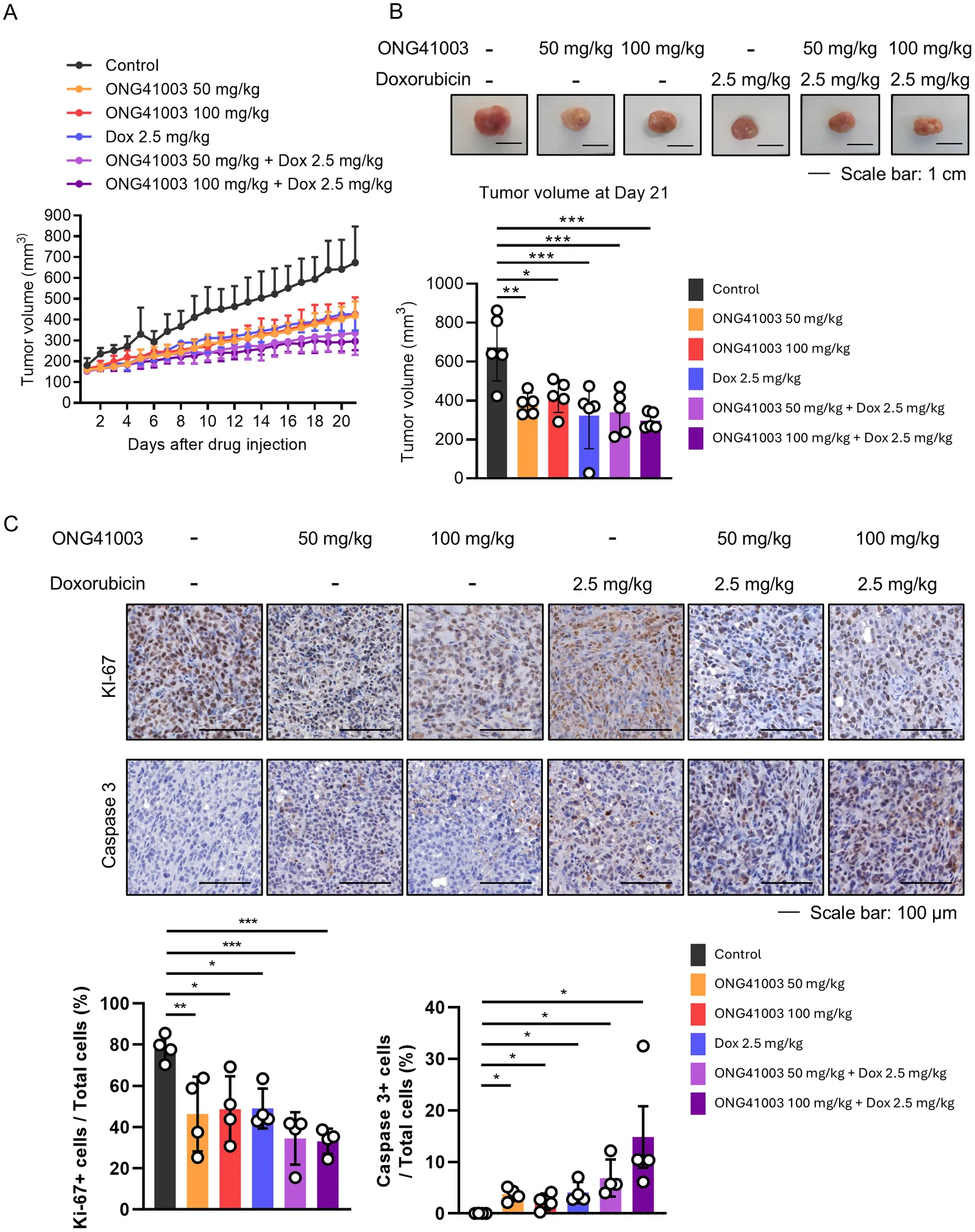
Synergistic effect of ONG41003 with doxorubicin *in vivo*. **(A)** Tumor growth curves of MDA-MB-231 xenograft-bearing Balb/c nude mice treated with vehicle control, ONG41003 (50 or 100 mg/kg daily), doxorubicin (2.5 mg/kg weekly), or the indicated combination treatments for 21 days. Tumor volume was measured daily and calculated using the formula: tumor volume = length × width²/2. Data are presented as mean ± SEM (n = 5 mice per group). **(B)** Representative gross images of excised tumors collected at day 21 after treatment (top). Quantification of tumor volumes at the experimental endpoint is shown in the lower panel. Scale bar = 1 cm. **(C)** Immunohistochemical analysis of Ki-67 and cleaved caspase-3 expression in xenograft tumor tissues. Representative staining images are shown in the upper panels. Quantification of Ki-67-positive and caspase-3-positive cells was performed using InForm image analysis software in three randomly selected fields per tumor sample. Ki-67 staining was used to assess proliferative activity, whereas cleaved caspase-3 staining was used to evaluate apoptosis. Scale bar = 100 μm. *p < 0.05, **p < 0.01, ***p < 0.001.

Given the amphiphilic and micelle-forming properties of Gelucire 44/14, ONG41003 with more flexible and extended hydrophobic substituents is expected to exhibit improved solubilization compared to ONG41008 with more compact derivatives. Therefore, we chose the ONG41003 for *in vivo* studies. To evaluate the *in vivo* antitumor efficacy of ONG41003 and its potential synergistic interaction with doxorubicin, we employed mouse xenograft models. The data showed that ONG41003 (50 or 100 mg/kg) or doxorubicin (2.5 mg/kg) treatment with resulted in a significant reduction in tumor growth (**Figures 7A, B**). Notably, combined treatment with ONG41003 and doxorubicin led to a markedly greater reduction in tumor volume than that achieved by either ONG41003 or doxorubicin alone (**Figures 7A, B**). No obvious adverse events, including anorexia, salivation, diarrhea, vomiting, polyuria, anuria, or fecal changes, were observed throughout the treatment period. Histopathological examination of the liver and kidney revealed no apparent tissue damage, and serum biochemical parameters, including ALT, AST, creatinine, and BUN, remained within the normal range, indicating no detectable hepatic or renal toxicity following ONG41003 or combination treatment (**Supplementary Figure 1**). Individual tumor growth and body weight profiles for each mouse are presented in **Supplementary Figure 2**. Histopathological examination of xenograft mouse model tissues revealed that proliferating Ki67+ cells within the tumor were significantly reduced by ONG41003 or doxorubicin, and these cells were nearly completely eliminated by the combinatory treatment of them (**Figure 7C**). Additionally, the apoptotic cell number within the primary tumor was increased by ONG41003 or doxorubicin alone, and more importantly, apoptotic rates were further enhanced by ONG41003 treatment in combination with doxorubicin (**Figure 7C**). Collectively, these findings demonstrate that ONG41003 not only exhibits robust antitumor activity *in vivo* but also significantly enhances the therapeutic efficacy of doxorubicin without inducing overt toxicity. This dual impact on tumor cell proliferation and apoptosis highlights the potential of ONG41003 as a promising combination partner for conventional chemotherapy in the treatment of aggressive breast cancers.

## 4 Discussion

In this study, we evaluated the anti-cancer efficacy of the novel Eupatilin derivatives ONG41008 and ONG41003 in TNBC models. Both compounds inhibited cell proliferation, migration, and invasion while inducing cell-cycle arrest and apoptosis. Importantly, ONG41008 and ONG41003 not only exerted intrinsic antitumor activity but also enhanced the therapeutic efficacy of doxorubicin through synergistic interactions, accompanied by increased intracellular and intratumoral doxorubicin accumulation and attenuation of doxorubicin-induced ABCB1 expression. Given the pronounced heterogeneity and therapeutic challenges of TNBC, these findings highlight the therapeutic potential of both derivatives, with ONG41003 showing overall superior antitumor efficacy and favorable *in vivo* activity.

Eupatilin has been widely studied for its anti-inflammatory and cytoprotective properties and has shown modest anti-cancer effects in several tumor types (15). However, its direct application as an anti-cancer agent has been limited by relatively weak potency and unfavorable pharmacological characteristics. By modifying amine-containing alkoxy side chains into the flavone scaffold, ONG41008 and ONG41003 retain the core structure of Eupatilin while exhibiting substantially improved anti-proliferative activity in multiple TNBC cell lines (**Figure 1**). Notably, ONG41003 demonstrated consistent cytotoxicity across diverse TNBC models, suggesting that it exerts potent anti-proliferative effects across different TNBC cell lines regardless of their specific subtypes (**Figure 2**). This consistent cytotoxicity is particularly relevant given the pronounced inter- and intra-tumoral heterogeneity characteristic of TNBC. While ONG41008 exhibited limited cytotoxicity across the tested TNBC cell lines, ONG41003 demonstrated markedly enhanced and consistent anti-proliferative activity (**Figure 2**). These findings suggest that specific side-chain modifications can substantially influence the anti-tumor efficacy of Eupatilin-derived compounds.

Previous studies have demonstrated that Eupatilin modulates cell cycle progression and key signaling pathways governing cellular proliferation and growth (15, 16). Specifically, Eupatilin suppresses endometrial cancer cell proliferation by inducing G2/M phase arrest and modulating ERK and AKT phosphorylation (17). In glioma cells, Eupatilin inhibits proliferation, migration, and invasion while inducing cell cycle arrest at the G1/S transition (18, 19). Similarly, in esophageal cancer cells, Eupatilin attenuates cell growth through inhibition of AKT and ERK signaling and induction of G1 phase arrest (20). Consistent with these reports, ONG41008 and ONG41003 induced G2/M phase arrest and apoptosis in TNBC cells (**Figure 3**). Interestingly, however, both derivatives increased the phosphorylation of PI3K, AKT, and AMPK, indicating a signaling profile distinct from that previously reported for Eupatilin (**Figure 4E**). Although PI3K/AKT activation is generally associated with cell survival, the derivatives simultaneously induced apoptotic cell death. These findings may therefore reflect context-dependent or compensatory cellular responses to treatment. However, the observed phosphorylation changes alone do not establish ONG41008 and ONG41003 as multi-kinase inhibitors. Given the established role of PI3K/AKT signaling in breast cancer progression and heterogeneity (21), further mechanistic studies, including time-course analyses, direct kinase activity assays, and pathway-specific functional experiments, will be necessary to clarify how these signaling alterations contribute to the anticancer effects of ONG41008 and ONG41003 in TNBC.

ONG41008 and ONG41003 exhibited evidence of synergistic interactions with doxorubicin, whereas no synergistic effect was observed in combination with paclitaxel (**Figure 5**). This drug-specific synergy may be attributable to the distinct mechanisms of action of doxorubicin and paclitaxel. Doxorubicin primarily exerts its anticancer effects through DNA intercalation, inhibition of topoisomerase II, and the induction of DNA double-strand breaks, ultimately triggering apoptosis, whereas paclitaxel disrupts microtubule dynamics and induces mitotic arrest (22–24). Given these differences, it is plausible that ONG41003 and ONG41008 preferentially cooperate with DNA damage–based cytotoxic stress rather than mitotic interference. Importantly, the enhanced interaction with doxorubicin was accompanied by increased intracellular doxorubicin accumulation in TNBC cells and greater intratumoral accumulation in xenograft tumors. Because ATP-binding cassette (ABC) transporters play central roles in anthracycline efflux and chemoresistance, we further examined the expression of ABCB1 and ABCG2. Real-time PCR analysis demonstrated that doxorubicin treatment increased ABCB1 expression, whereas co-treatment with ONG41003 or ONG41008 significantly attenuated this induction. In contrast, ABCG2 expression was not significantly affected by combination treatment. Taken together, these findings suggest that doxorubicin induces an adaptive increase in ABCB1 expression, thereby promoting drug efflux and limiting intracellular drug retention. ONG41003 and ONG41008 appear to partially reverse this response, which may contribute to the increased intracellular accumulation and enhanced antitumor efficacy of doxorubicin. Because the present study evaluated transporter expression only at the mRNA level, further studies examining ABCB1 protein expression and functional efflux activity will be required to establish the causal relationship between ABCB1 suppression and enhanced doxorubicin accumulation. However, because transporter expression was evaluated only at the mRNA level, additional studies examining protein expression and functional efflux activity will be necessary to confirm this mechanism. These findings suggest that ONG41003 and ONG41008 may act as rational combination partners that selectively amplify the therapeutic efficacy of anthracycline-based chemotherapy. Nevertheless, further mechanistic studies are warranted to elucidate how these compounds modulate the tumor cell context to favor doxorubicin activity, and to determine whether similar synergistic interactions extend to other classes of DNA-damaging agents. Additionally, the synergistic interaction between ONG41003 or ONG41008 and doxorubicin raises the possibility that therapeutic efficacy could be maintained while reducing the required dose of doxorubicin. Such a dose-reduction strategy may potentially mitigate the dose-limiting toxicities commonly associated with anthracycline-based chemotherapy, including cardiotoxicity and systemic adverse effects. Consistent with the *in vitro* findings, combination treatment with ONG41003 and doxorubicin produced greater tumor growth inhibition than either monotherapy in the TNBC xenograft model, accompanied by reduced Ki-67 expression and increased apoptotic cell death. Moreover, no detectable systemic toxicity was observed, as evidenced by the absence of histopathological abnormalities and normal serum biochemical parameters, supporting the favorable safety profile of the combination treatment. While the *in vivo* dosing of ONG41003 (50–100 mg/kg) used in this study appears higher than that of conventional cytotoxic agents such as doxorubicin, this may partly reflect the broader therapeutic window generally associated with polyphenol-derived compounds. Based on FDA guidelines for body surface area normalization, the *in vivo* dose of 50–100 mg/kg in mice corresponds to an estimated human equivalent dose (HED) of approximately 4.1–8.1 mg/kg. Nevertheless, we acknowledge that Eupatilin derivatives, including ONG41003, may face pharmacokinetic limitations related to poor aqueous solubility and limited systemic bioavailability. Although the use of an amphiphilic vehicle (Gelucire 44/14) improved compound solubilization in the present study, further optimization of formulation strategies, such as nanoparticle encapsulation or lipid-based delivery systems, will likely be required to enhance systemic exposure and support future clinical translation.

Collectively, our findings suggest that ONG41003 enhances the efficacy of doxorubicin not only through its intrinsic antitumor activity but also by promoting intracellular drug retention, potentially via attenuation of ABCB1-mediated drug efflux. Although further studies are needed to reveal the precise mechanisms underlying their molecular and phenotypic activities, ONG41008 and ONG41003 may serve as potential therapeutic strategies to improve clinical outcomes in TNBC.

## Data Availability

The original contributions presented in the study are included in the article/**Supplementary Material**, further inquiries can be directed to the corresponding author.
